# Control of anisotropy of a redox-active molecule-based film leads to non-volatile resistive switching memory†
†Electronic supplementary information (ESI) available: Experimental details (materials and methods), device properties (crystal structure, GIWAXS, retention, FE-SEM, UV-vis spectra, and IR spectra), control experiment using TPHAP, theoretical calculations and crystallographic details. CCDC 1888177. For ESI and crystallographic data in CIF or other electronic format see DOI: 10.1039/c9sc04213j


**DOI:** 10.1039/c9sc04213j

**Published:** 2019-10-17

**Authors:** Jaejun Kim, Hiroyoshi Ohtsu, Taizen Den, Krittanun Deekamwong, Iriya Muneta, Masaki Kawano

**Affiliations:** a Department of Chemistry , School of Science , Tokyo Institute of Technology , 2-12-1 Ookayama, Meguro-ku , Tokyo 152-8550 , Japan . Email: mkawano@chem.titech.ac.jp; b Department of Electrical and Electronic Engineering , School of Engineering , Tokyo Institute of Technology , 4259 Nagatsuta-cho, Midori-ku , Yokohama 226-8503 , Kanagawa , Japan

## Abstract

Control of the π–π interaction direction in a redox-active π-molecule based film led to the formation of new mechanistic nonvolatile resistive switching memory: a redox-active organic molecule, 2,5,8-tri(4-pyridyl)1,3-diazaphenalene, showed non-volatile bistable resistance states with a high on-off ratio, retention, and endurance only when the molecular orientation was anisotropic. Control experiments using redox-active/redox-inert organic molecules with isotropic/anisotropic molecular orientations implied that the formation of conductive oxidized π–π stacking layers from non-conductive neutral π–π stacking layers is responsible for resistive switching phenomena, indicating new mechanisms such as ReRAM. Our findings will give a comprehensive understanding of electron transport in organic solid materials based on the effects of redox-activity and molecular arrangement, leading to fabrication of a new class of ReRAM based on organic molecules.

## Abstract 2

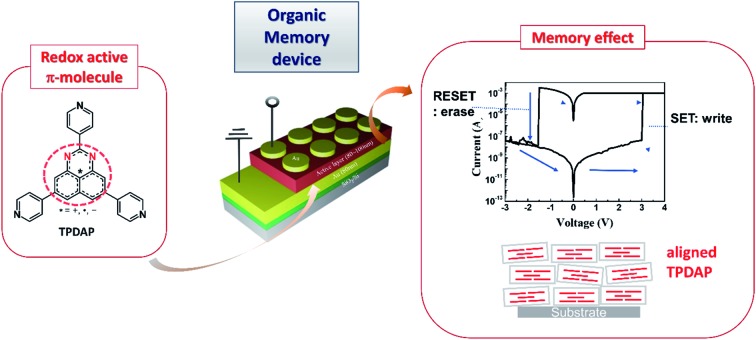
Control of the π–π interaction direction in a redox-active π-molecule based film led to the formation of new mechanistic non-volatile resistive switching memory.

## Introduction

**Fig. 1:**
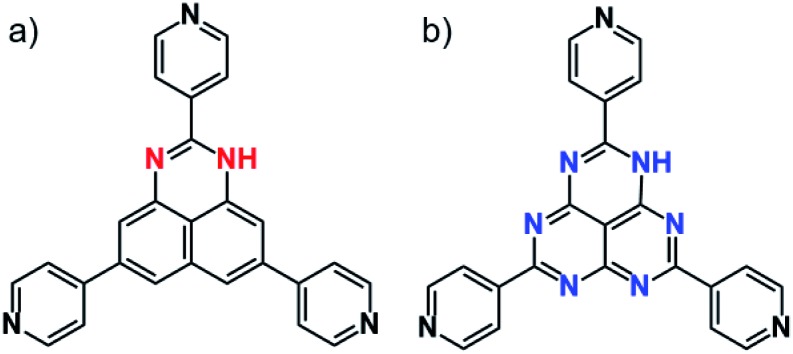
Molecular structures of redox-active (a) TPDAP and redox-inert (b) TPHAP.

Control of molecular arrangement in the solid state can be performed using intermolecular interactions such as π–π interactions, hydrogen bonding, and dispersion force. In particular, π–π interactions in large π-molecules play a crucial role in constructing functional molecular materials.^1^–^4^ When the π–π distance is close (in the range of 3.3 to 3.6 Å) to exchange electrons (or holes), the π–π stacking column can act as an electron transfer path. In such cases, it is expected to utilize the π–π stacking materials for functional electronic devices.^5^,^6^ As one of the large π-conjugated molecules, we reported a redox active DAP (1,3-diazaphenalene) derivative (Fig. 1a).^7^,^8^ This molecule is comprised of a redox active DAP skeleton^9^,^10^ and three pyridyl moieties so that it can be used to form redox active coordination networks. Indeed, self-assembly of this molecule with Cd^2+^ produces a π–π columnar structure of DAP to create an electron transfer path only when the molecule is partially oxidized.^8^ This fact encouraged us to utilize the DAP derivative for further applications. Because the oxidation of the Cd coordination network increases electron conductivity, it indicates that the conductive and non-conductive (insulating) properties in the solid state can be switched by redox state change (applying voltage) using an anisotropic π–π columnar electron transfer path of DAP. This idea inspired us to utilize them as memory devices, so-called resistive switching memory devices (ReRAM).

Over the last decade, ReRAM have been intensively studied not only in academia but also in the industrial field because of expectations for replacing ordinary memory devices owing to their simple architecture, scalability, CMOS compatibility, and their fast operation.^11^–^17^ However, the development of ReRAM faces the limit of commercialization because of the unstable switching mechanism ascribed to metallic filament formation through the defects in the active layer which was hardly controlled.^18^ Although various switching mechanisms were proposed, they still remain ambiguous because of lack of the detailed mechanistic study.^19^–^23^ To overcome these difficulties, we need a paradigm shift to utilize molecular properties themselves instead of utilizing filament formation from electrodes composed of the device.

Herein, we report general guidelines for designing resistive switching memory devices using redox-active organic molecules, highlighting the importance of redox-activity and anisotropic molecular arrangement without filament formation. A redox-active organic molecule, 2,5,8-tri(4-pyridyl)1,3-diazaphenalene (TPDAP; Fig. 1a)^7^ showed non-volatile bistable resistance states with a high on–off ratio, retention, and endurance only when the molecular orientation in thin film was anisotropic. Control experiments using redox-active/redox-inert organic molecules (Fig. 1) with isotropic/anisotropic molecular orientations implied that the formation of conductive oxidized π–π stacking layers from non-conductive neutral π–π stacking layers is responsible for resistive switching phenomena. Our findings will give a comprehensive understanding of electron transport in organic solid materials based on the effects of redox-activity and molecular arrangement.

Redox-active organic molecules can play a promising role as flexible electronic elements because the electronic state can be readily controlled by external energy.^24^,^25^ Although various redox-active organic molecules were synthesized, attempts to apply them to electronic devices have been limited because the proper molecular arrangement in the solid state in addition to their redox-activity must be considered.^26^ For example, redox-active organic molecules have an appropriate HOMO–LUMO level to generate additional energy states by oxidation or reduction; however, it sometimes does not have an appropriate electron transfer path with neighbouring molecules because of poor orbital overlapping.^8^ In addition, chemical instability of redox-active organic molecules is a barrier to practical applications.^27^,^28^ Control of the redox state with consideration of the molecular arrangement and its chemical stability in the solid state can be a key to the application of redox-active organic molecules to electronic devices. In this sense, an anisotropic TPDAP π–π columnar structure is ideal for electronic devices with an electron transfer pathway caused by DAP and high stability.^8^

The TPDAP film showed two stable resistance states modulated by an external voltage with a high on/off ratio, retention time and endurance. TPDAP, a derivative of the graphene fragment but having totally different physical properties from graphene, has a large π-plane of a redox-active central skeleton with three pyridine rings, inducing the insulating in-plane layer and possibly conductive out-of-plane layer in their π–π stacking structure. The film with anisotropic orientation was initially non-conductive (off-state) but converted to be conductive (on-state) owing to the appropriate redox potential of TPDAP by applying voltage. Grazing incidence wide angle X-ray scattering (GIWAXS) and UV-vis/IR spectroscopic studies of the on- and off-states indicated that the resistance modulation did not result from the change of structure/electronic state/chemical bonds at the bulk level. As a control experiment, an isotropic TPDAP film was formed which showed a linear *I*–*V* relationship, indicating no memory effect. Furthermore, an anisotropic film of redox-inert 2,5,8-tri(4′-pyridyl)-1,3,4,6,7,9-hexaazaphenalene (TPHAP, Fig. 1b)^29^ having the same molecular shape as TPDAP did not show resistive switching even though it had the same π–π stacking structure as that of TPDAP. These facts verified that the anisotropically oriented layers *via* π–π stacking and its oxidation/reduction process promoted two reversible resistance states. In other words, the external voltage reversibly generated a conductive oxidized layer in the local region from the non-conductive neutral layer. These results clearly support the importance of molecular arrangement in the solid state and its redox activity for designing various electronic devices and resistive switching memory devices.

## Results and discussion

### Thin film device formation

**Fig. 2:**
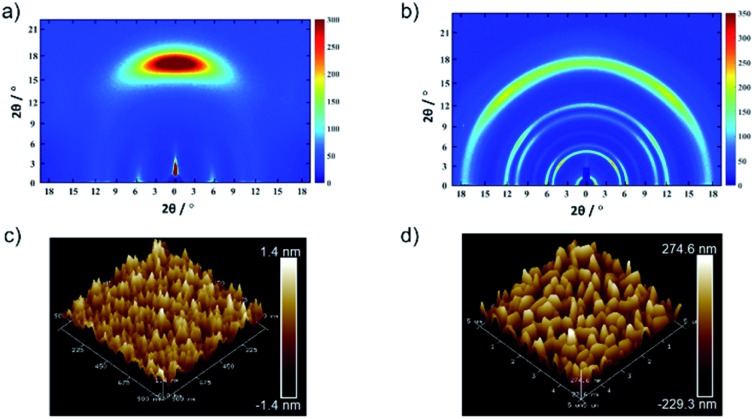
Molecular arrangements and microstructures of a TPDAP film: GIWAXS of (a) aniso-TPDAP and (b) iso-TPDAP. AFM images of (c) aniso-TPDAP with *R*_a_ = 0.33 nm and (d) iso-TPDAP with 82.6 nm. The detailed peak assignment from the 1D cut of in-/out-of-plane of each film is available in the ESI (see Fig. S1–S3†).

An anisotropic TPDAP film (aniso-TPDAP) and an isotropic TPDAP film (iso-TPDAP) were selectively prepared by thermal evaporation at different substrate temperatures, 25 °C and 80 °C, respectively. While GIWAXS of aniso-TPDAP showed one broad peak in the out-of-plane and three peaks in the in-plane (Fig. 2a), that of iso-TPDAP showed five kinds of ring-like diffraction (Fig. 2b). Because single crystals suitable for single crystal diffraction analysis were grown under the same conditions as the film deposition (Fig. S1†), each diffraction from GIWAXS was clearly assigned in comparison with the single crystal X-ray structure (Fig. S2 and S3†). The 1D cut of out-of-plane from GIWAXS of aniso-TPDAP showed a broad peak of 2*θ* = 17.1° (1 0 2) corresponding to a π–π stacking distance (3.69 Å). Although the broadness of the peak indicated a deviation of the π–π distance and a slightly inclined lamination, the anisotropic orientation was confirmed. In addition, the 1D cut of in-plane showed three peaks of 2*θ* = 5.7° ((0 2 0) and (0 0 2)), 2*θ* = 10.4° (0 4 0), and 2*θ* = 11.82° (0 0 4), indicating that the periodicity was formed by intermolecular hydrogen bonding among TPDAPs. In other words, the hydrogen bonding of N···H among TPDAPs played a crucial role in maintaining the graphene-like in-plane structure, and the large π-plane made them stacked through π–π interactions in aniso-TPDAP as shown in Fig. S1.† These interactive features of TPDAP complementarily contributed to the formation of a highly uniform surface with a roughness average (*R*_a_) of 0.33 nm observed by atomic force microscopy (AFM) (Fig. 2c). In contrast with aniso-TPDAP, the 1D cut of iso-TPDAP regardless of the orientation showed all five sharp peaks that appeared in aniso-TPDAP, indicating that the substrate temperature induced randomly oriented polycrystalline growth (Fig. 2b). The AFM image of iso-TPDAP revealed that the polycrystalline phase was grown with an *R*_a_ of 82.6 nm (Fig. 2d). The large π-plane and multi-intermolecular interactivity produced the highly packed structure that is the same as the single crystal structure, although their orientation depended on the substrate temperature. It is noteworthy that the time dependent spin density calculation from electron spin resonance (ESR) measurement of single crystals of TPDAP showed that only the surface was oxidized by air, which means that the highly packed structure of TPDAP could prevent the bulk oxidation in air.^7^ Such chemical stability of a redox-active organic molecule in the solid state is quite important to use it for practical applications such as electronic devices. The retention of TPDAP molecules by thin film formation was confirmed by IR and UV (Fig. S7, S9 and S10†) of the film which can be assigned as TPDAP molecules from the reported literature.^7^ Furthermore, the PXRD pattern of GIWAXS matches the predicted pattern of single crystal X-ray data (Fig. S2†), indicating no decomposition by deposition of Au.

### Memory effects using the TPDAP thin film

**Fig. 3:**
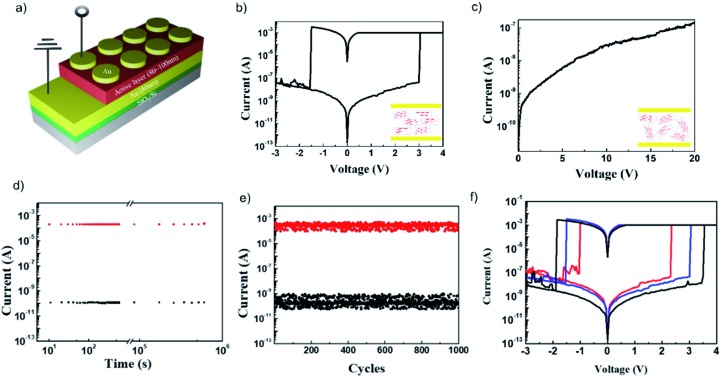
Resistive switching memory device: (a) device architecture of resistive switching memory. (b) *I*–*V* characteristic of Au/aniso-TPDAP (100 nm)/Au/SiO_2_/Si and (c) Au/iso-TPDAP (80 nm)/Au/SiO_2_/Si. (d) Retention measurement of Au/aniso-TPDAP (100 nm)/Au/SiO_2_/Si performed with a reading bias of –0.1 V under ambient conditions. (e) Endurance measurement of Au/aniso-TPDAP (100 nm)/Au/SiO_2_/Si performed with a reading bias of –0.1 V at the interval time between set and reset. (f) Thickness dependent *I*–*V* characteristic of Au/aniso-TPDAP/Au/SiO_2_/Si. Each thickness was confirmed by scanning electron microscopy (SEM; see Fig. S5†).

The current–voltage (*I*–*V*) characteristics of the sandwiched device, Au/aniso-TPDAP/Au/SiO_2_/Si (Fig. 3a), showed non-volatile resistive switching memory behaviour with a high on–off ratio, retention, and endurance, which was not observed in the device Au/iso-TPDAP/Au/SiO_2_/Si (Fig. 3b and c). The positive voltage sweep was first applied to Au/aniso-TPDAP/Au/SiO_2_/Si that initially was a high resistance state (HRS, or an off-state), and then the current rapidly increased to *I*_cc_, a low resistance state (LRS, or an on-state), when the voltage reached the set voltage (*V*_set_) as a set (write) process. The LRS was retained when the voltage was turned off or during positive sweeps, which was considered as a non-volatility of resistive switching. The device returned to the HRS when a negative voltage sweep reached the reset voltage (*V*_reset_) as a reset (erase) process. The resistances of both the HRS and LRS were retained without any degradation over 10^5^ seconds without applying voltage (Fig. 3d), that is, they can be used for the data storage. This retention ability was also stable at 120 °C that was normally considered as a maximum operation temperature in computational devices (Fig. S4†). In addition, the set/reset process could be repeated over 10^3^ cycles as a rewritable memory device (Fig. 3e). Thickness dependent *I*–*V* characteristics with 65 nm, 100 nm, and 130 nm (thickness was confirmed by cross-sectional FE-SEM; Fig. S5†) exhibited that higher thickness has higher resistance, and both *V*_set_ and *V*_reset_ also increased as the thickness was increased (Fig. 3f). It should be noted that in the case of metallic filament formation grown by electrochemical reactions on an electrode such as Cu, Pt, and Ag, *V*_reset_ should be independent of the thickness of the active layer, and only *V*_set_ increased as the thickness was increased, which is not the case in this study. In addition, Au/aniso-TPDAP/Au/SiO_2_/Si did not require the forming process that was typically involved in the electrochemical growth on an electrode. Notably, in contrast to aniso-TPDAP, Au/iso-TPDAP/Au/SiO_2_/Si did not exhibit the resistive switching even when voltage was applied up to 20 V, which means that randomly oriented molecular arrangement could not have a possible conductive pathway (Fig. 3c). In addition, the *I*–*V* characteristic of in-plane TPDAP measured using the aniso-TPDAP film deposited on a pre-patterned Au electrode did not show resistive switching with almost negligible current (Fig. S6†). These results indicate that the switching mechanism of Au/aniso-TPDAP/Au/SiO_2_/Si was only related to changes of physical properties in the active layer and the anisotropic column of π–π stacking is the only possible electron/hole transport pathway in the TPDAP films.

GIWAXS, UV-vis and IR spectroscopic studies of on- and off-states of the memory device indicated that the resistance modulation did not result from the change of structure/electronic state/chemical bonds at the bulk level. A 5 nm Au top electrode was deposited over the active layer to minimize the influence of the electrode during the measurement, and an electrode of 80 nm was deposited on a portion of pre-deposited Au for the contact area of an analyser tip (Fig. S7†). The highly p-doped Si, ITO glass, and Au/SiO_2_/Si were used as a bottom electrode for GIWAXS, UV-vis and IR spectroscopy, respectively. GIWAXS of Au/aniso-TPDAP/p-Si of the on-/off-state was performed to compare the structural difference, which showed that the π–π distance of the on-state (3.61 Å) was slightly smaller than that of the off-state (3.64 Å) (Fig. S8†). Although the exact cause of the small difference could not be elucidated, one possible reason could be that the oxidized species of a part of TPDAPs induced a better interaction among layers as compared with neutral layers. Both UV-vis spectroscopy of Au/aniso-TPDAP/ITO/glass and IR spectroscopy of Au/aniso-TPDAP/Au/SiO_2_ showed little difference between on- and off-states (Fig. S9 and S10†). These results indicate that the resistive switching phenomenon was not caused by a change at the bulk level, but perhaps on the localized region. It was also confirmed by the cell size dependent *I*–*V* characteristics that the resistance of the HRS decreased as the cell size increased, whereas the resistance of the LRS was constant, indicating that the localized conductive pathway was generated in the LRS (Fig. S11†).^30^

### Control experiments using TPHAP

**Fig. 4:**
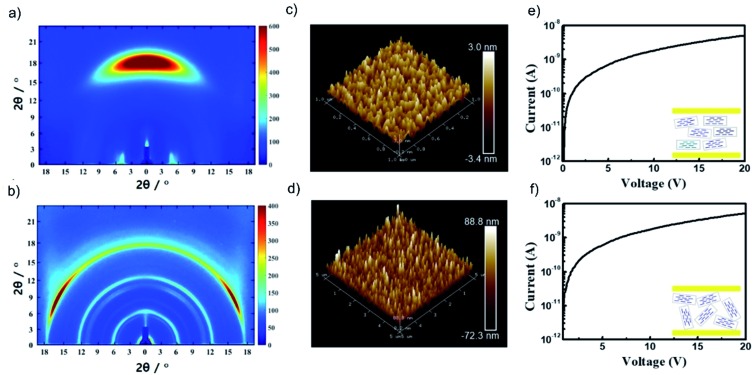
Control experiments using redox-inert TPHAP: GIWAXS of (a) aniso-TPHAP and (b) iso-TPHAP. AFM images of (c) aniso-TPHAP with *R*_a_ = 0.71 nm and (d) iso-TPHAP with *R*_a_ = 17.3 nm. *I*–*V* characteristic of (e) Au/aniso-TPHAP (100 nm)/Au/SiO_2_/Si and (f) Au/iso-TPHAP (100 nm)/Au/SiO_2_/Si. The detailed peak assignment from the 1D cut of in-/out-of-plane of each film is available in the ESI (see Fig. S13–S15†). Each thickness was confirmed by SEM (Fig. S16†).

A redox-inert organic molecule, TPHAP, having the same molecular shape as TPDAP, was also used in the device fabrication as a control experiment to confirm the role of the redox-activity. The neutral protonated form of TPHAP (denoted as ‘TPHAP’ below) was synthesized from the potassium form of TPHAP anions to avoid the influence of ion conductivity on a device (Fig. S12†).^31^ While GIWAXS of aniso-TPHAP showed one broad peak on the out-of-plane and two peaks on the in-plane (Fig. 4a), that of iso-TPHAP showed 5 kinds of ring-shaped diffraction (Fig. 4b). Each peak from the 1D cut of in-/out-of-plane was well assigned using the single crystal structure of protonated TPHAP (Fig. S13–S15†). Aniso-TPHAP had a highly oriented structure in which π–π stacking was in a direction perpendicular to the substrate with an *R*_a_ of 0.71 nm (Fig. 4c), and on the other hand, iso-TPHAP had randomly oriented molecular arrangement with an *R*_a_ of 17.3 nm (Fig. 4d). The tendency of molecular arrangement depending on the experimental conditions was consistent with that of TPDAP, so that TPHAP could be used in the control experiment. The *I*–*V* characteristics of both Au/aniso-TPHAP/Au/SiO_2_ and Au/iso-TPHAP/Au/SiO_2_ showed a typical plot of ohmic contact without any resistive switching phenomena (Fig. 4e and f). This clearly shows that molecular redox activity is required to obtain memory effects.

### Resistive switching mechanism

**Fig. 5:**
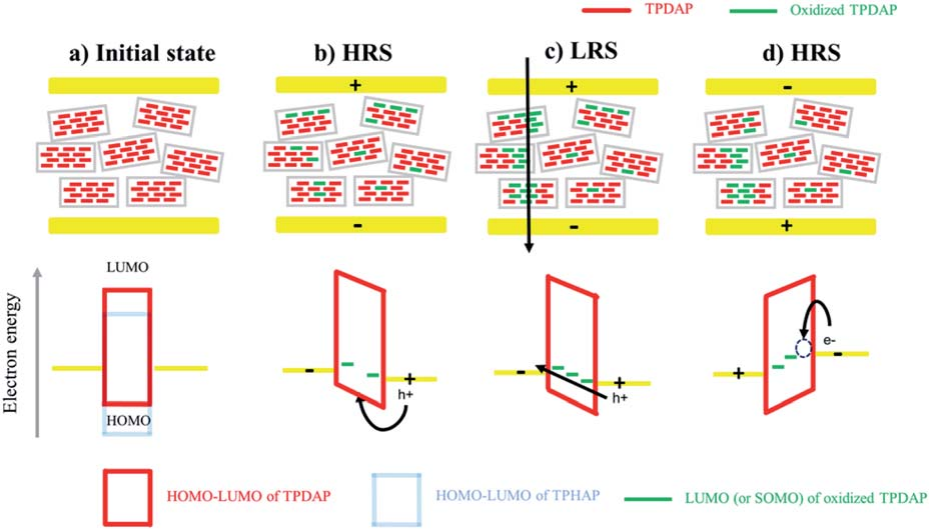
Resistive switching mechanism: schematic of the resistive switching mechanism and its band diagram. (a) The HOMO level of TPDAP was lower than that of TPHAP. The hole injection barrier between the Au electrode and TPDAP was small enough to inject holes. (b) When positive voltage was applied to the top electrode, holes were injected into TPDAP, while they could not be injected into TPHAP. The injected holes could be trapped in redox-active TPDAP, resulting in its oxidation. (c) When the oxidized species were connected between a top electrode and a bottom electrode, a conductivity pathway was generated only in aniso-TPDAP, but not in iso-TPDAP. (d) When negative voltage was applied to a top electrode and reached *V*_reset_, the oxidized TPDAP could be reduced by the accumulated electrons injected by the top electrode on the surface of TPDAP.

On the basis of the above experimental results, the resistive switching mechanism is proposed using an energy diagram (Fig. 5). The HOMO–LUMO levels of TPDAP and TPHAP were calculated by the DFT method with the B3LYP/6-31G* level of theory (Fig. S17†). When the positive voltage was applied to the Au top electrode, holes could be injected into the TPDAP film, and a part of TPDAP was oxidized. In fact, because the surface of TPDAP was chemically oxidized in air, the oxidized species was already generated on the surface of TPDAP.^7^ This was the reason why Au/aniso-TPDAP/Au/SiO_2_/Si did not require the forming process. The further oxidation process created the region beyond Ohm's law only in *I*–*V* characteristics of aniso-TPDAP and iso-TPDAP, which indicated that TPDAP acted as a charge trapping site (Fig. S18†).^32^ When oxidized TPDAP constituted a chain that connected the top and bottom electrodes along the π–π stacking, which was only possible in the case of aniso-TPDAP, the current rapidly increased in the LRS. On the other hand, although iso-TPDAP had oxidized species, randomly oriented π–π stacking did not allow the construction of a conductive path for them. The in-plane measurement of *I*–*V* characteristic in the aniso-TPDAP film was evident for the anisotropic conductivity of TPDAP films as discussed above. The theoretical calculation of hole mobility using Amsterdam Density Functional (ADF) theory revealed that poor orbital overlapping in the in-plane direction induced the poor hole mobility of 6.51 × 10^–6^ cm^2^ V^–1^ S^–1^ and π–π interaction resulted in a possible conduction pathway with 0.53 cm^2^ V^–1^ S^–1^ (Fig. S19†).^33^,^34^ As a reset process in aniso-TPDAP, when negative voltage was applied to the top electrode and reached *V*_reset_, electrons were injected into the oxidized TPDAP film, following the reduction of the oxidized TPDAP to TPDAP, resulting in the rupture of the conductive chain as a HRS. In contrast to TPDAP, because TPHAP has a hole injection barrier that is too high to be oxidized, the possible conductive chain was not constructed regardless of their molecular arrangements which are proper or not. In other words, anisotropically oriented layers *via* π–π stacking with an appropriate redox potential for the oxidation/reduction process promoted two reversible resistance states.

## Conclusions

Aniso-TPDAP having π–π stacking aligned out-of-plane with a highly uniform surface owing to its multi-interactivity showed the resistive switching phenomenon. GIWAXS, UV-vis and IR spectroscopy of on-/off-states and control experiments of iso-TPDAP and redox-inert TPHAP revealed that the resistive switching mechanism resulted from the formation/rupture of aligned oxidized TPDAP chains. In this study, we provide general guidelines for the design of resistive switching memory devices using redox-active organic molecules, highlighting the importance of molecular arrangement and appropriate redox potential.

## Conflicts of interest

There are no conflicts to declare.

## Supplementary Material

Supplementary information

Crystal structure data
