# Supplementary material for: Control of anisotropy of a redox-active molecule-based film leads to non-volatile resistive switching memory
Source: Chem Sci. 2019 Oct 17;10(47):10888–93. doi: 10.1039/c9sc04213j (PMC7066666; doi:10.1039/c9sc04213j)
Supplement: Supplementary file 1 [file SC-010-C9SC04213J-s001.pdf]

Electronic Supplementary Material (ESI) for Chemical Science.
This journal is © The Royal Society of Chemistry 2019

Electronic Supplemental Information

Control of anisotropy of redox-active molecule-based film leads to non-volatile resistive switching memory

Jaejun Kim,[a] Hiroyoshi Ohtsu,*[a] Taizen Den,[a] Krittanun Deekamwong,[a] Iriya Muneta,[b] and Masaki Kawano *[a]

[a] *Department of Chemistry, School of Science, Tokyo Institute of Technology, 2-12-1 Ookayama, Meguro-ku, Tokyo 152-8550, Japan*

[b] *Department of Electrical and Electronic Engineering, School of Engineering, Tokyo Institute of Technology, 4259 Nagatsuta-cho, Midori-ku, Yokohama 226-8503, Kanagawa, Japan*

**Corresponding author: M. Kawano, e-mail: mkawano@chem.titech.ac.jp*

Contents

Figures and Tables

Fig. S1. Single crystal X-ray structure of TPDAP.

Fig. S2. Assignment of diffractions in GIWAXS of aniso-TPDAP.

Fig. S3. Assignment of diffractions in GIWAXS of iso-TPDAP.

Fig. S4. Retention measurement of Au/aniso-TPDAP (100 nm)/Au/SiO_2/Si measured with a reading bias of -0.1 V at 120 °C under ambient conditions.

Fig. S5. Cross-sectional FE-SEM images of aniso-TPDAP and iso-TPDAP films.

Fig. S6. a) Schematic illustration of device architecture for in-plane I-V measurement of aniso-TPDAP b) in-plane I-V characteristic of aniso-TDPAP

Fig. S7. Schematic illustration of sample preparation for GIWAXS, UV-vis, and vibrational measurement of on- and –off state.

Fig. S8. GIWAXS measurement of Au/aniso-TPDAP/p-doped Si of on- and off-state.

Fig. S9. Vibrational spectroscopic measurements of aniso-TPDAP of on- and off- state.

Fig. S10. UV spectroscopic measurements of aniso-TPDAP of on- and off- state.

Fig. S11. Cell size dependent I-V characteristics of Au/aniso-TPDAP/Au/SiO_2/Si.

Fig. S12. Synthesis of protonated TPHAP.

Fig. S13. Single crystal X-ray structure of protonated TPHAP.

Fig. S14. Assignment of diffractions in GIWAXS of aniso-TPHAP.

Fig. S15. Assignment of diffractions in GIWAXS of iso-TPHAP.

Fig. S16. Cross-sectional FE-SEM images of a) aniso-TPHAP and b) iso-TPHAP.

Fig. S17. HOMO-LUMO energy levels of TPDAP and TPHAP.

Fig. S18. Double-log plot of I-V characteristic of Au/aniso-TPDAP/Au/SiO_2/Si device.

Fig. S19. Theoretical calculation for hole mobility in TPDAPs.

References for ESI

Materials and Methods

Materials

All reagents were purchased from commercial sources and used without further purification. TPDAP was synthesized by 5 steps as described in our previous report.[S1(7)] 2,5,8-tri(4’-pyridyl)-1,3,4,6,7,9-hexaazaphenalene (TPHAP) was synthesized from the potassium form of TPHAP prepared by the method in our previous report.[S2(29)] A solution of potassium form of TPHAP in 2M HCl was precipitated by adding acetone to obtain a protonated form, then crystalline powder was sublimed at 450 °C to obtain a neutral form of TPHAP. ^{1}H NMR (400 MHz, DMSO-d6) δ 8.46 (*d*, 6H, J = 6.05 Hz), 8.86 (*d*, 6H, J = 6.05 Hz); Anal. Calcd for C22H14.32N9O0.66: C, 63.63; H, 3.48; N, 30.35. Found: C, 63.54; H, 3.19; N, 30.05. The X-ray crystal structure was determined using synchrotron radiation (see supporting. cif file and Fig. S13).

X-ray diffraction analysis

GIWAXS experiments were performed at the 9A beamline in Pohang Accelerator Laboratory (PAL) using the monochromatized X-ray radiation source of 11.035 keV with a sample-to-detector distance of 221.227 mm. Scattering data were typically collected for 8-10 s using a two-dimensional (2D) charge-coupled detector (model Rayonix 2D Mar, Evanston, IL, USA). The incidence angle (α_i) was set at 0.12° which typically was between the critical angle of an organic film and a silicon substrate. The scattering angles were corrected according to the position of the X-ray beam calibrated with a standard sample. 1D cuts of in-plane and out-of-plane direction were profiled from the 2D scattering pattern. The d-spacing of diffraction was calculated with $d = n\lambda/2\sin\theta$. The single crystal of TPHAP was obtained by sublimation at 380 °C in an ampule sealed under 10^{-2} torr. The diffraction data for a single crystal of protonated TPHAP was recorded on a Rigaku Varimax diffractometer with Saturn system equipped with a Rigaku GNNP low temperature device using graphite-monochromated Mo *K*α radiation (wavelength = 0.71075 Å). The diffraction images were processed using the CrysAlis^{pro} software. The structures were solved by the direct method (SHELXT-2015) and refined by full-matrix least squares calculations on F^2 (SHELXL-2015) using the SHELX-TL program package.[S3] C44H26N18, *Mr* = 802.79, crystal dimensions 0.01 × 0.03 × 0.04 mm^3, orthorhombic, *Pbca*, *a* =20.085(3) Å, *b* = 7.0453(9) Å, *c* = 23.461(3) Å, V = 3485.1(8) Å^3, *T* = −173 °C, *Z* = 4, ρ_{calcd} = 1.538 g cm^{-3}, μ = 0.1 mm^{-1}, 3094 unique reflections out of 2495 with $I > 2\sigma(I)$, 280 parameters, $2.598° < \theta < 25.054°$, R_1= 0.0659, wR_2= 0.1752, GOF = 0.932. CCDC deposit number 1888177.

Device fabrications

A 40 nm Au bottom electrode was deposited on SiO_2 (300 nm)/Si by thermal evaporation, then an active layer of TPDAP or TPHAP was also thermally deposited at 230 °C and 280 °C, respectively, under high vacuum (10^{-6} torr), followed by a 80 nm Au top electrode with 100 μm diameter was deposited on the active layer, resulting in the sandwich structure of electrode/active layer/electrode/SiO_2/Si. The molecular orientation of TPDAP and TPHAP films was controlled by substrate temperatures when an active layer was thermally deposited on the substrate. The anisotropically oriented films via π-π stacking (aniso-TPDAP and aniso-TPHAP) were obtained when the substrate temperature was kept at 25 °C, while isotropic films (iso-TPDAP and iso-TPHAP) were obtained at 80 °C.

Characterizations

An VEECO Dimension 3100 running with a Nanoscope V controller was used to obtain AFM images on Au/SiO_2/Si. The cross-sections of all films were captured using a high-resolution field emission SEM (JSM-7800F Prime, JEOL) with 5 kV acceleration voltage to confirm the thickness. The detailed sample preparation of on-/off-state of ansio-TPDAP for the IR, and UV-vis spectroscopic study were described in Fig. S7. The diffuse reflectance UV-vis spectra of on-/off-state of Au/iso-TPDAP/ITO/Glass were recorded on VersaSTAT 3, AMETEK. IR spectroscopy of on-/off-state of Au/iso-TPDAP/Au/SiO_2/Si was performed on a FT-IR spectrometer with the reflection mode (Varian 670). Current-voltage (I-V) characteristics of devices were measured on a semiconductor parameter analyzer (4200-SCS, Keithley and B1500A, Agilent).

Theoretical calculations

The HOMO-LUMO levels of TPDAP and TPHAP were calculated by the DFT method with the B3LYP/6-31G* level of theory based on the crystal structure. The transfer integrals and reorganization energy of TPDAP for mobility calculation was calculated by the DFT with B3LYP* functional and TZP basic set using the Amsterdam density functional (ADF) theory program.

The mobility was evaluated on the basis of the Marcus theory given by the following Einstein equations applied to the solid state,

$$\mu = \frac{\mathrm{e}}{\mathrm{k}_B \mathrm{T}}$$

Where e is the elementary charge and k_B is the Boltzmann constant. D is the diffusion coefficient of the carrier given by,

$$D = \frac{1}{2\mathrm{n}} \sum_i r_i k_i P_i \qquad P_i = \frac{k_i}{\sum_j k_j}$$

$$k = \frac{t^2}{\hbar} \sqrt{\frac{\pi}{\lambda \mathrm{k}_B \mathrm{T}}}\, e^{\left(-\frac{\lambda}{4\mathrm{k}_B \mathrm{T}}\right)}$$

N is the dimension (n=3), r_i is an intermolecular distance, and P_i is a relative probability of charge transport. The hopping rate (k) was calculated from the transfer integrals and reorganization energy for each transport pathway, where i is the number of transport pathways and h is the Plank constant.

The reorganization energy (λ) is a structural relaxation accompanied by the charge transfer such as $M^0 + M^{\pm} \rightarrow M^{\pm} + M^0$. The energies of each state are estimated from the adiabatic energy surfaces, and λ is given by the following equation:

$$\lambda = \lambda_1 + \lambda_2 = \left(E^*_{charged} - E_{charged}\right) + \left(E^*_{neutral} - E_{neutral}\right)$$

where E^* indicates the energy with the original geometry before the charge transfer.

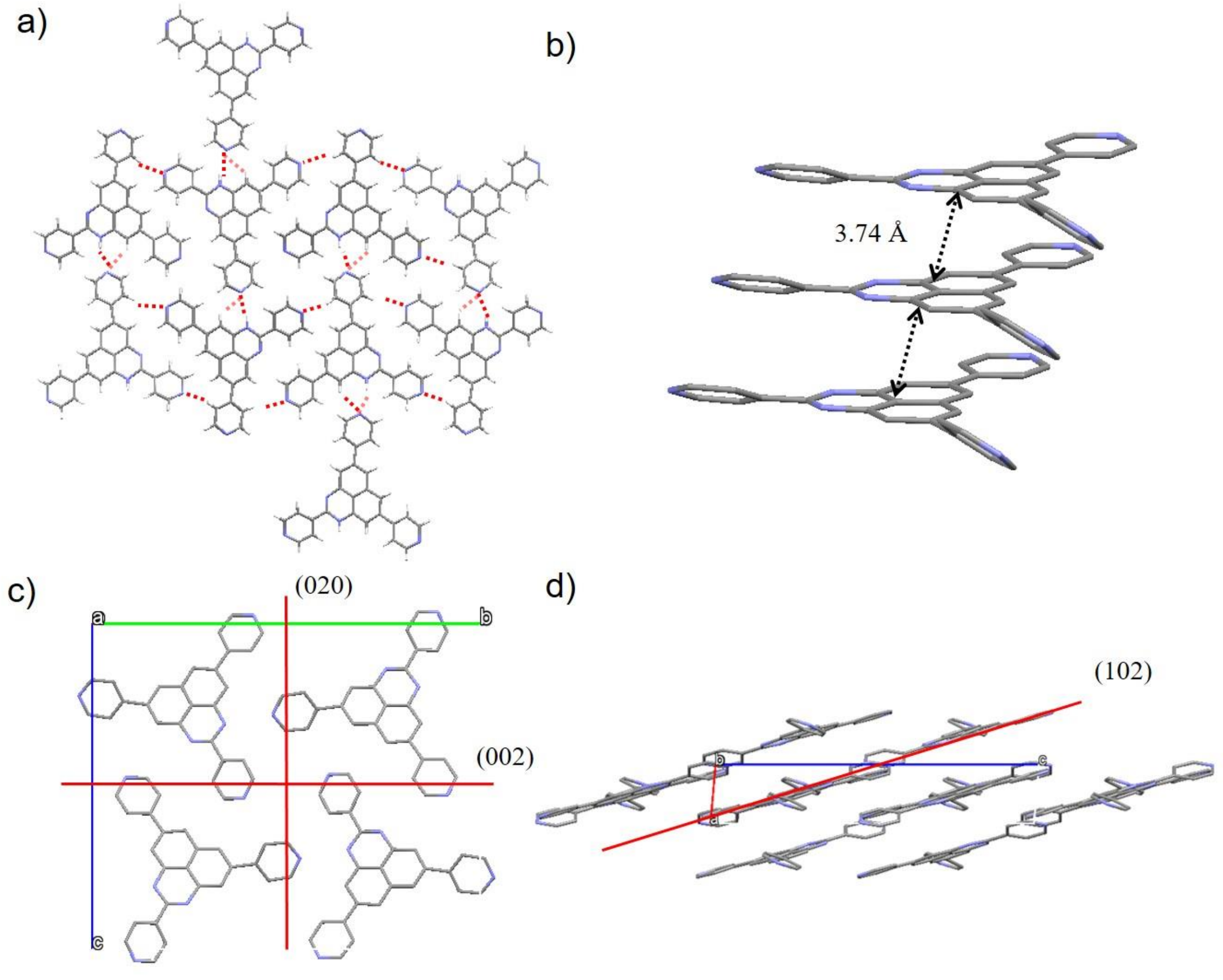

Fig. S1. Single crystal X-ray structure of TPDAP from the reference to clarify the Miller indices to explain GIWAXS.[S1] a) Intermolecular hydrogen bonding sites. B) π-stacking structure. c) Miller indices of (020) and (002) planes. d) Miller index of (102) plane. The planes were displayed by the Mercury 3.10. For clarity, disordered pyridine rings were removed. The hydrogen bonding of N···H among TPDAPs played a crucial role of maintaining the graphene-like in-plane structure, and the large π-plane made them stacked through π-π interactions in aniso-TPDAP. Gray, C; blue, N; white, H.

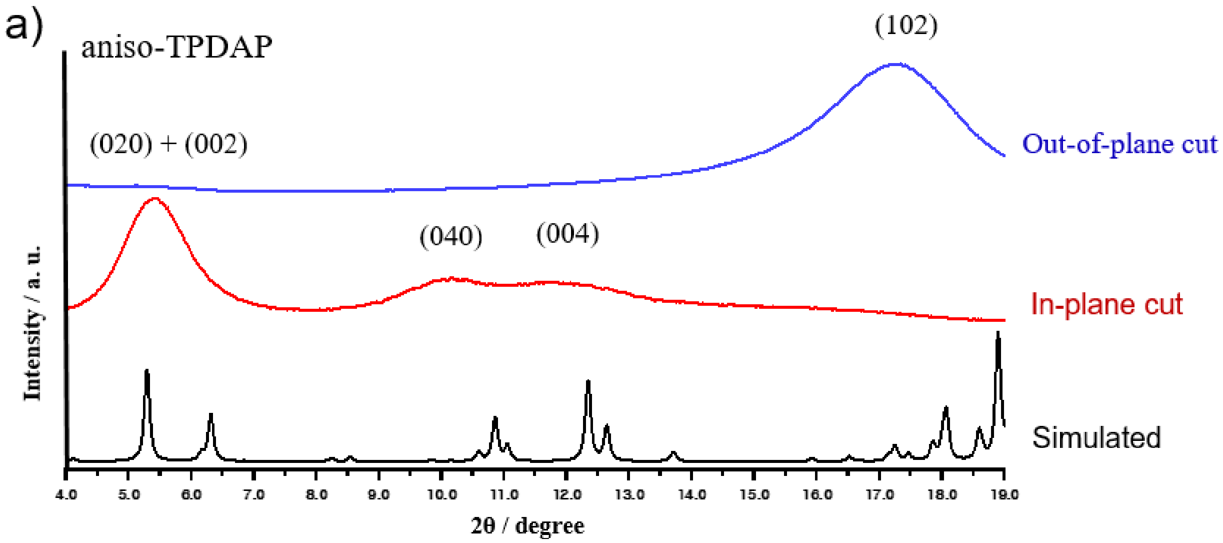

b)

Index	n	2θ (º)	d (Å)
(020) + (002)	1	5.42	11.88
(040)	2	10.03	12.85
(004)	2	11.81	10.92
(102)	1	17.81	3.63

Fig. S2. Assignment of diffractions in GIWAXS of aniso-TPDAP. a) 1D cut of aniso-TPDAP from 2D GIWAXS and a simulated pattern from the single crystal X-ray analysis. b) The table of peak assignments. The d-spacing of diffractions were calculated with $d = n\lambda/2\sin\theta$. Miller index was placed on the single crystal X-ray analysis: Monoclinic $P2_1/c$.

a)

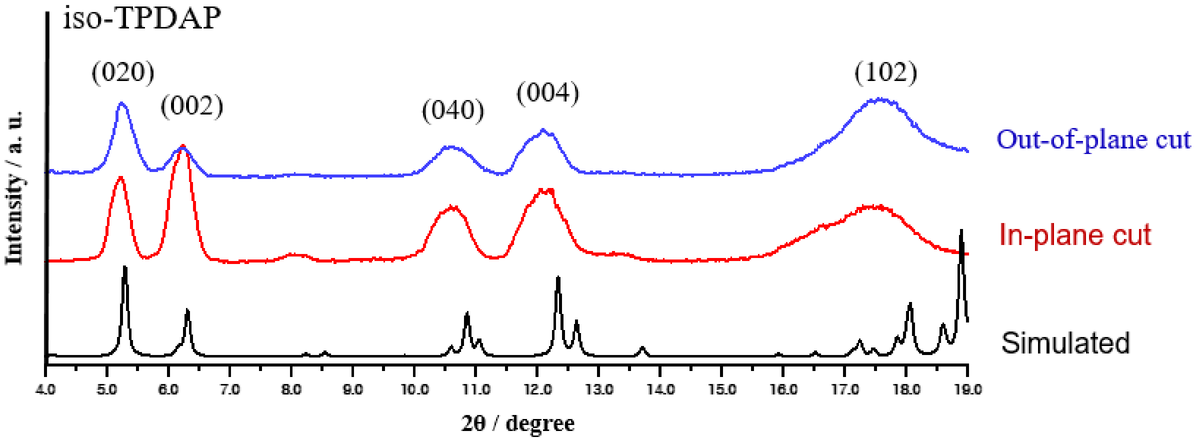

b)

Index	n	2θ	d (Å)
(020)	1	5.24	12.29
(002)	1	6.22	10.36
(040)	2	10.69	12.06
(004)	2	12.24	10.54
(102)	1	17.52	3.69

Fig. S3. Assignment of diffractions in GIWAXS of iso-TPDAP. a) 1D cut of iso-TPDAP from 2D GIWAXS and a simulated pattern from the single crystal X-ray analysis. b) The table of peak assignments. The d-spacing of diffractions were calculated with $d = n\lambda/2\sin\theta$. Miller index was placed on the single crystal X-ray analysis: Monoclinic $P2_1/c$.

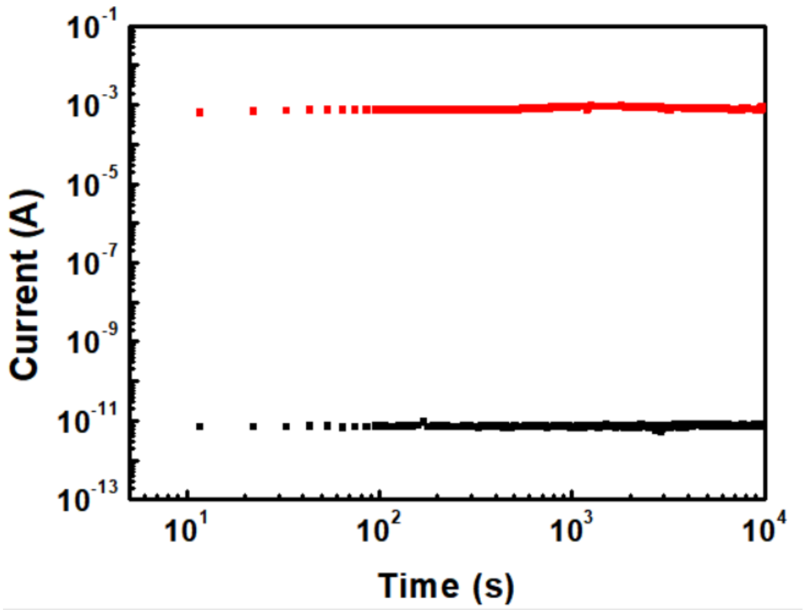

Fig. S4. Retention measurement of Au/aniso-TPDAP (100 nm)/Au/SiO_2/Si measured with a reading bias of -0.1 V at 120 °C under ambient conditions.

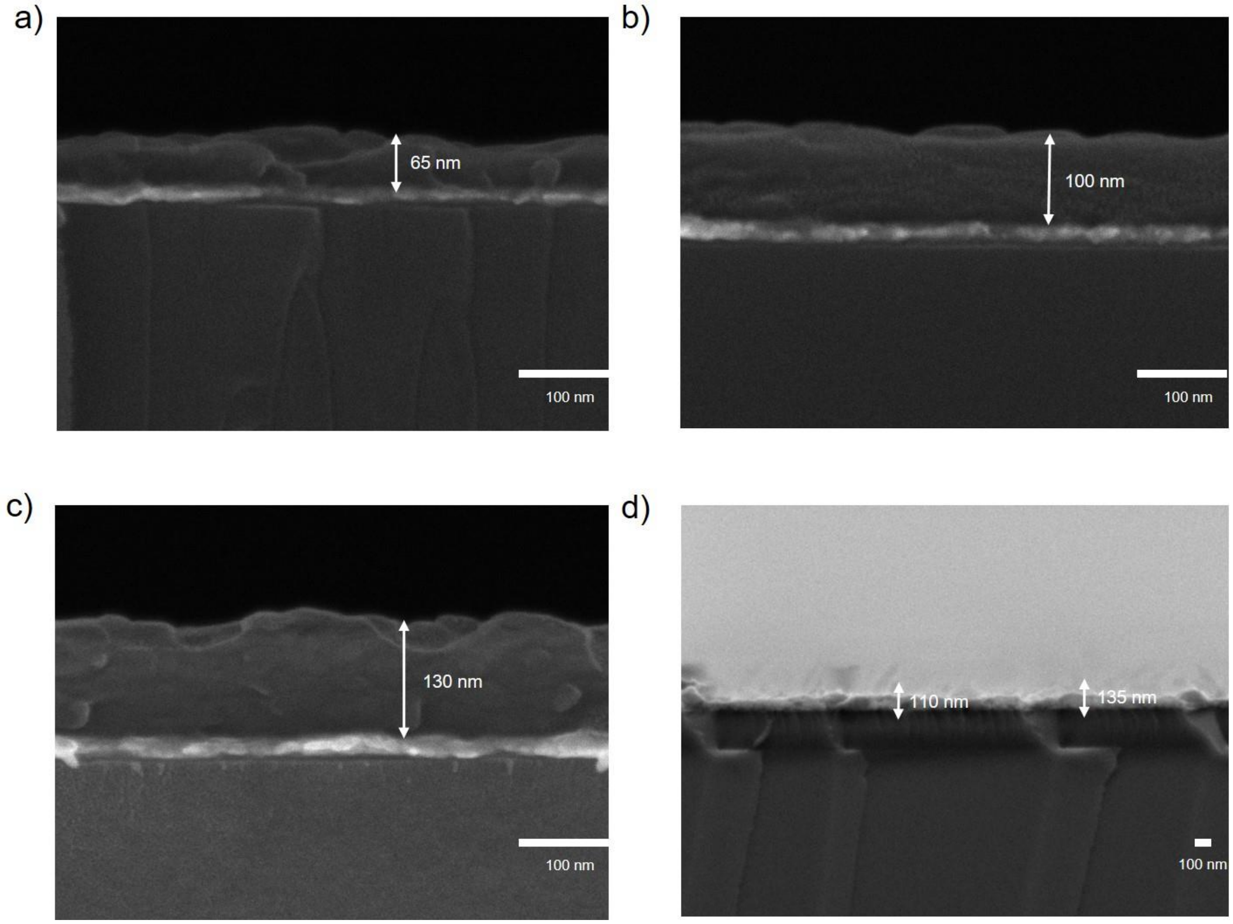

Fig. S5. Cross-sectional FE-SEM images of a, b, c) aniso-TPDAP and d) iso-TPDAP. The thickness of films used for the thickness dependent I-V characteristics of aniso-TPDAP were confirmed.

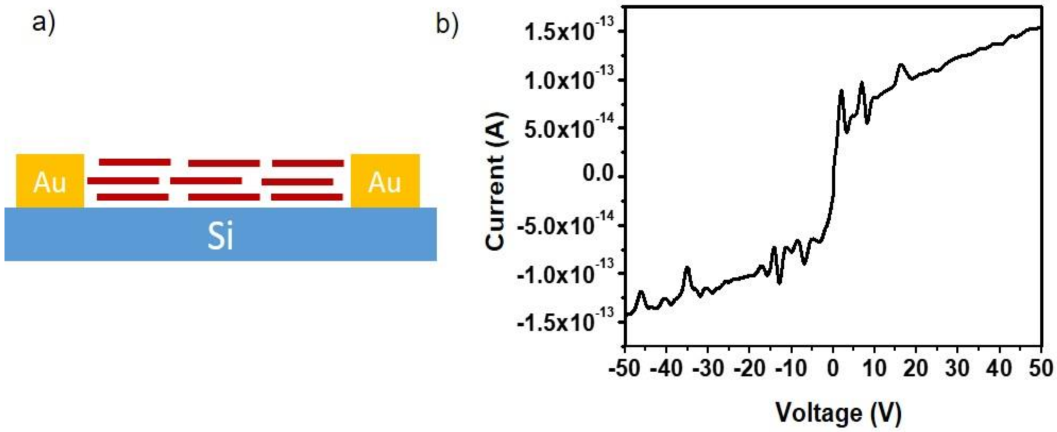

Fig. S6. a) Schematic illustration of device architecture for in-plane I-V measurement of aniso-TPDAP b) in-plane I-V characteristic of aniso-TDPAP

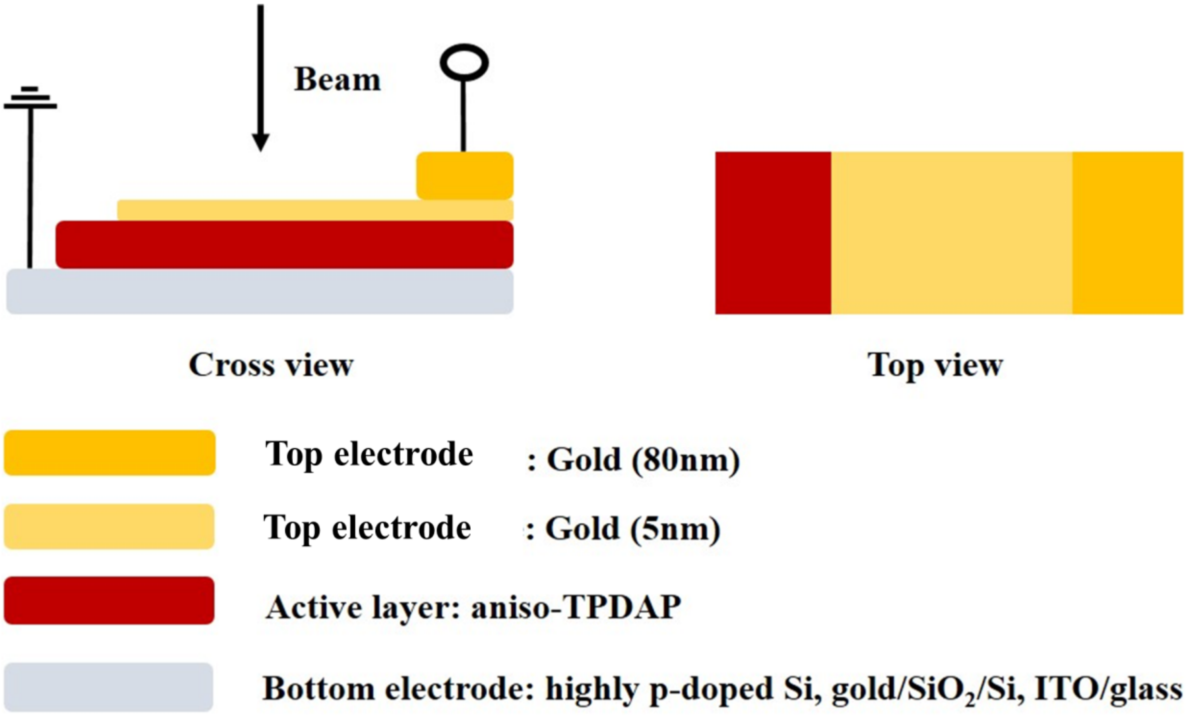

Fig. S7. Schematic illustration of sample preparation for GIWAXS, UV-vis, and vibrational measurement of on- and –off state. A 5 nm Au top electrode was deposited over the active layer to minimize the influence of electrode during the measurement, and a 80 nm electrode were deposited on a portion of pre-deposited Au for the contact area of an analyzer tip. The highly p-doped Si, ITO glass, and Au/SiO_2/Si were used as a bottom electrode for GIWAXS, UV-vis and IR spectroscopy, respectively.

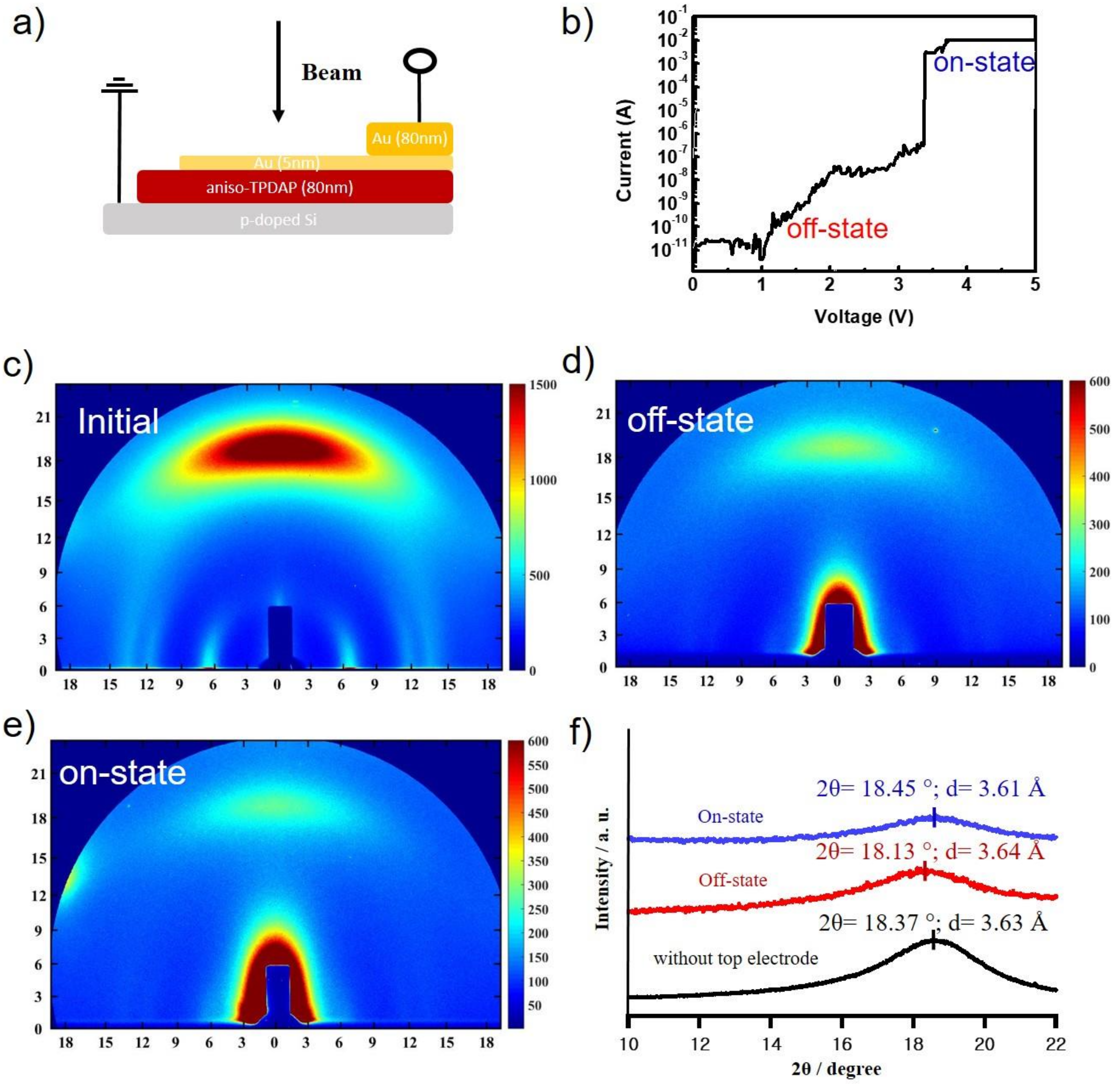

Fig. S8. GIWAXS measurement of Au/aniso-TPDAP/p-doped Si of on- and off-state. a) Schematic illustration of device architecture b) I-V characteristic of a device prepared for GIWAXS measurement c) The GIWAXS of c) the initial without a top electrode (aniso-TPDAP/p-doped Si) d) off-state e) on-state. f) 1D cut of out-of-plane in the initial without top electrode/off-state/on-state of samples. The peak on the out-of-plane corresponding to π–π distance was calculated. The slight increase of a π–π distance after top electrode deposition might come from the thermal effect from the Au particle during deposition. Although the exact cause of the small difference between the off-state and on-state could not be elucidated, one possible reason could be that the oxidized species of a part of TPDAPs induced better interaction among layers as compared with neutral layers.

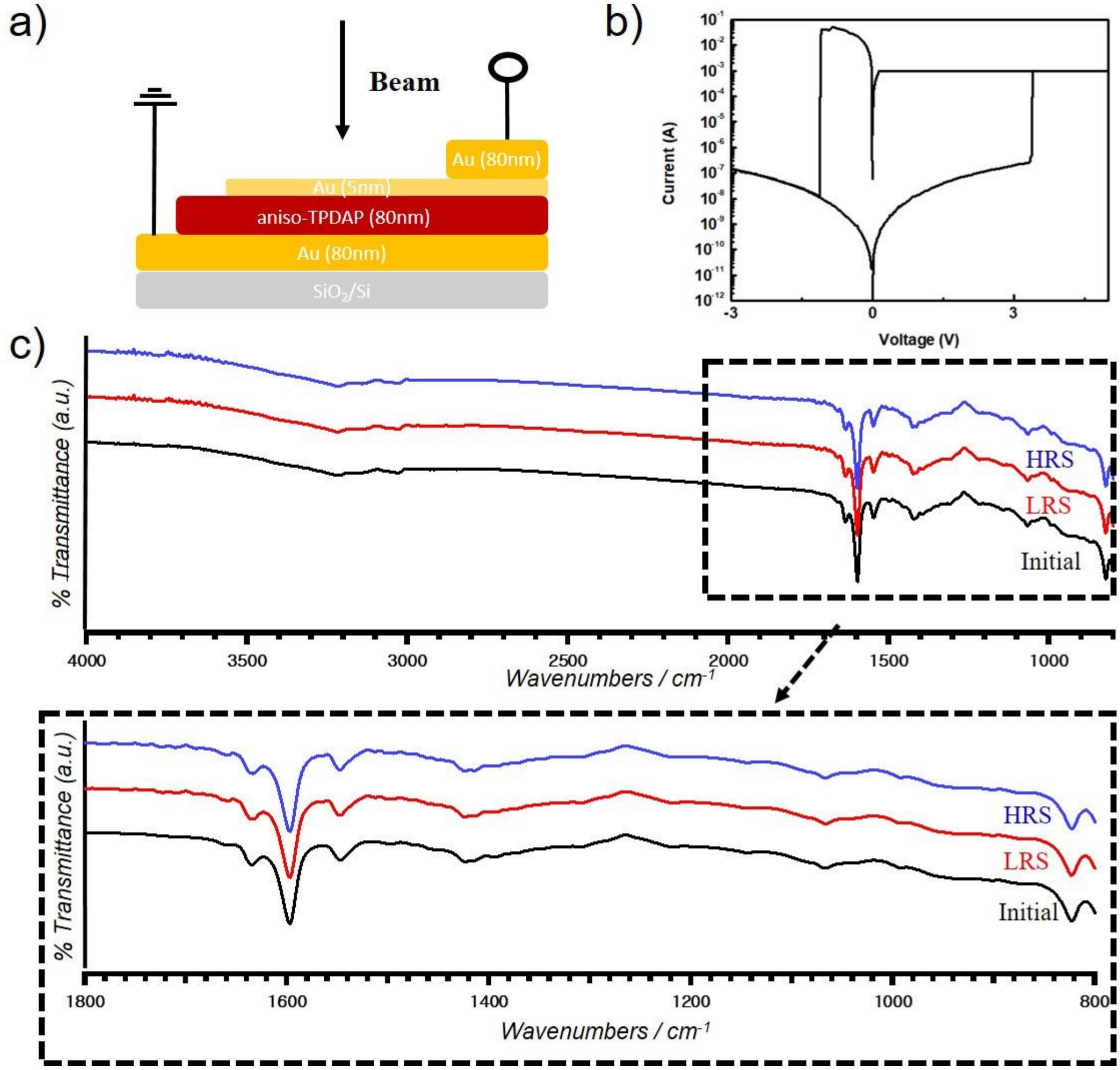

Fig. S9. Vibrational spectroscopic measurements of aniso-TPDAP of on- and off- state. a) Schematic illustration of device architecture b) I-V characteristic of device prepared for vibrational spectroscopic measurement c) Vibrational spectroscopic measurement of aniso-TPDAP of on- and off- state. The significant difference was not observed between LRS and HRS except for the peak corresponding to water. The measurement was performed under vacuum.

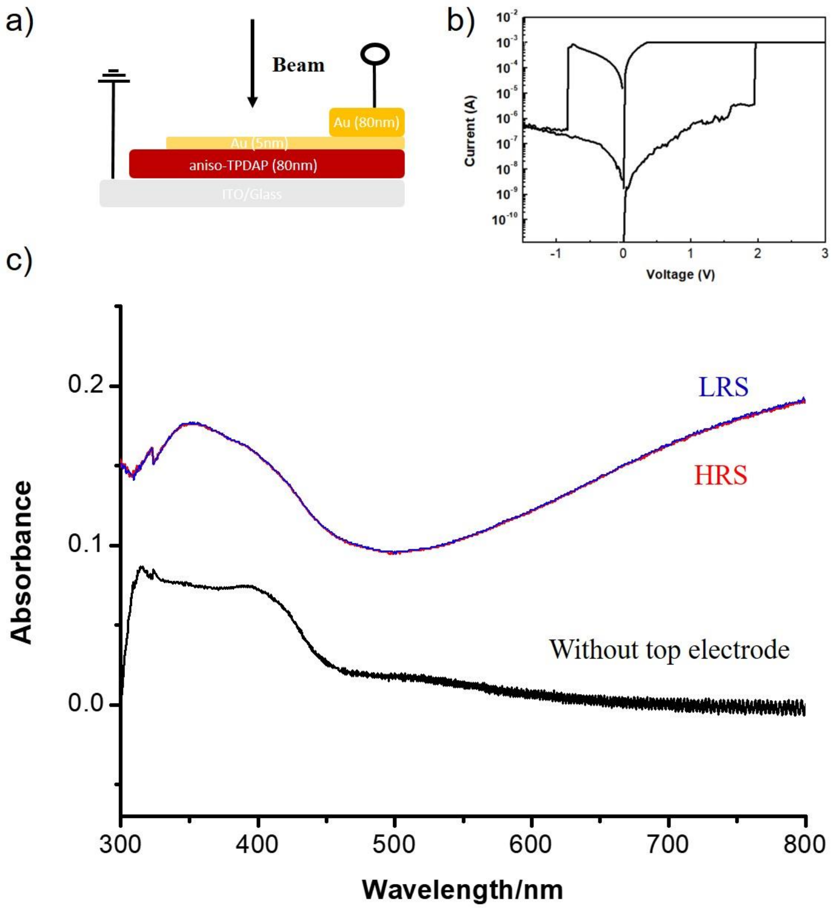

Fig. S10. UV spectroscopic measurements of aniso-TPDAP of on- and off- state. a) Schematic illustration of device architecture b) I-V characteristic of a device prepared for UV spectroscopic measurement c) UV spectroscopic measurement of aniso-TPDAP of on- and off- state. The significant difference was not observed between LRS and HRS. The measurement was performed under ambient conditions.

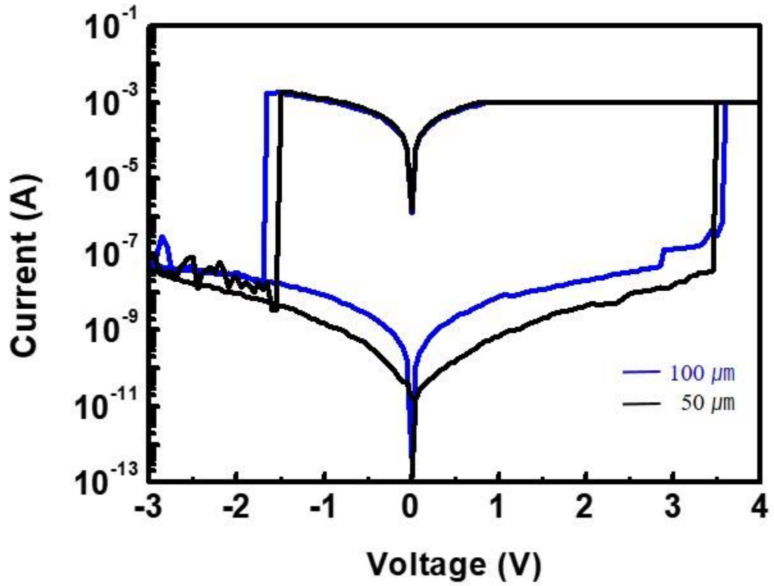

Fig. S11. Cell size dependent I-V characteristics of Au/aniso-TPDAP/Au/SiO_2/Si. Each diameter of cells was 50 μm (black) and 100 μm (blue), respectively. The resistance of the HRS decreased as the cell size increased, whereas the resistance of LRS was constant, indicating that the localized conductive pathway was generated in LRS.

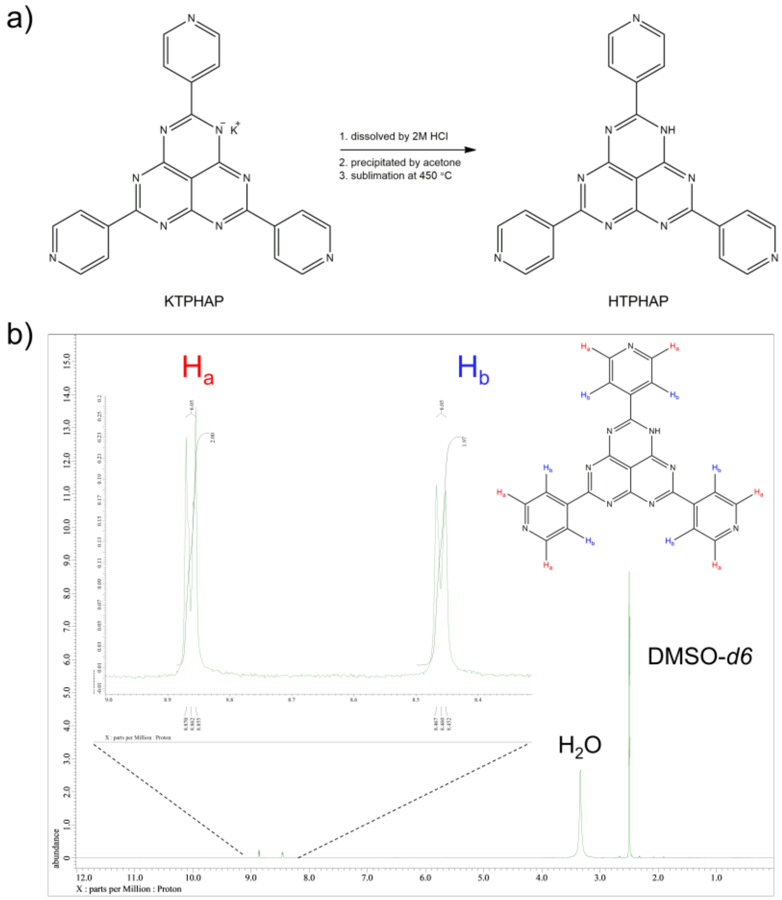

Fig. S12. Synthesis of protonated TPHAP. a) The reaction scheme of protonated TPHAP. b) ^{1}H nuclear magnetic resonance spectrum of protonated TPHAP in DMSO-d6.

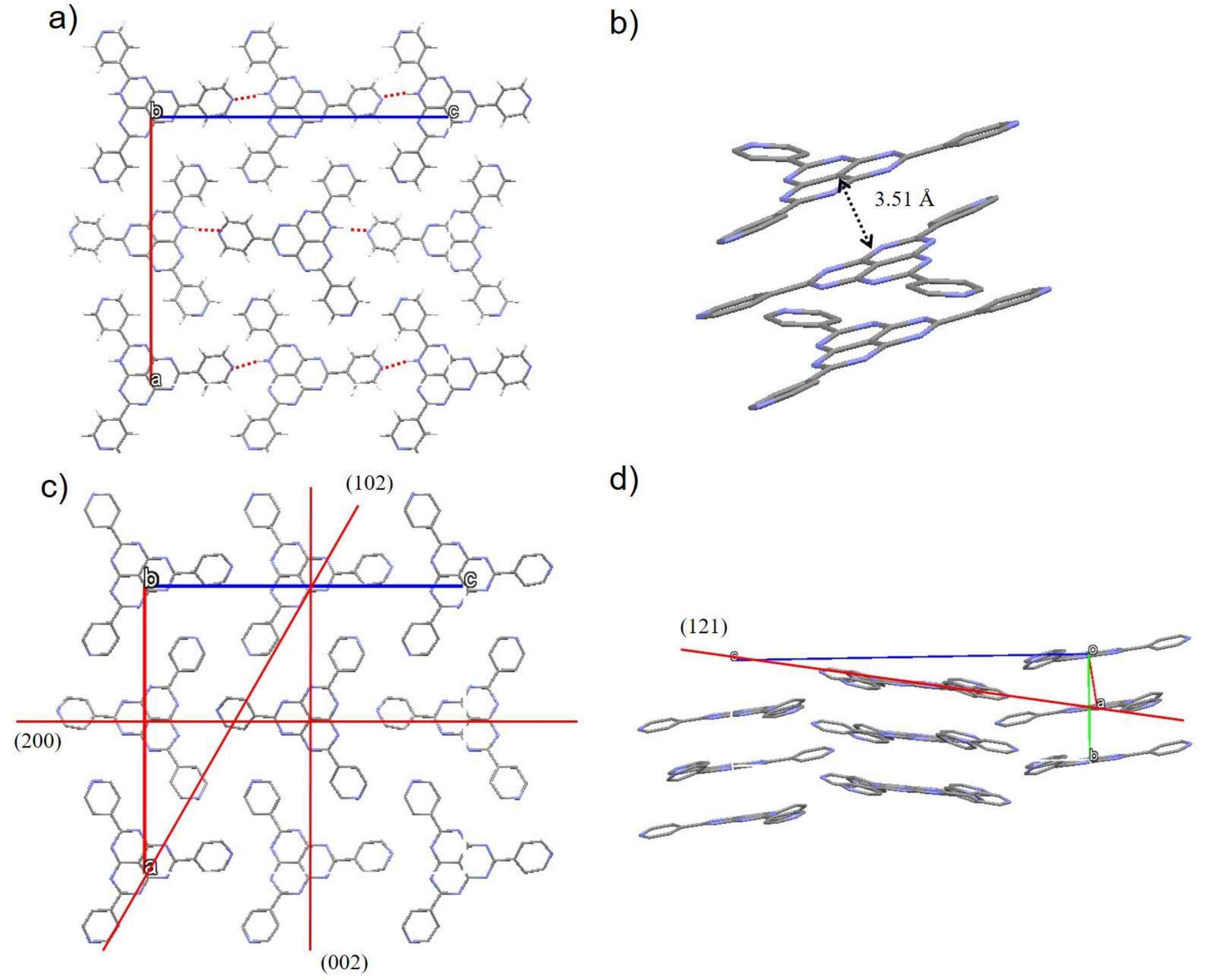

Fig. S13. Single crystal X-ray structure of protonated TPHAP. a) Intermolecular hydrogen bonding sites. B) π-stacking structure. c) Miller indices of (200), (002), and (102) planes. d) Miller index of (121). The planes were displayed by the Mercury 3.10. Similarly to the packing structure of aniso-TPDAP, the hydrogen bonding of N···H among TPHAPs played a crucial role of maintaining the graphene-like in-plane structure, and the large π-plane made them stacked through π-π interactions in aniso-TPHAP. Gray, C; blue, N; white, H.

a)

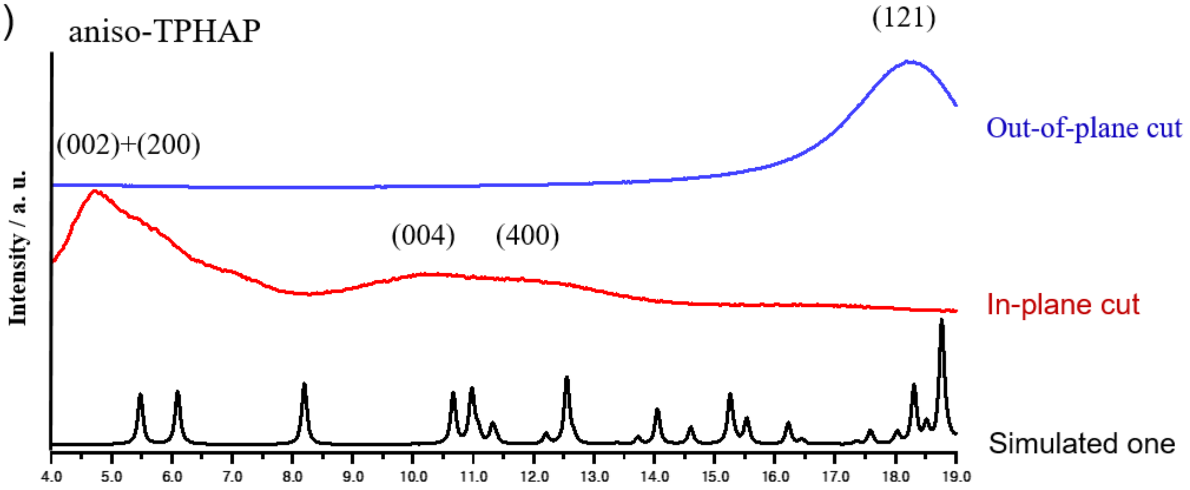

b)

Index	n	2θ (°)	d (Å)
(002) + (200)	1	4.81	13.35
(004)	2	10.18	12.62
(400)	2	11.81	10.88
(102)	1	18.26	3.53

Fig. S14. Assignment of diffractions in GIWAXS of aniso-TPHAP. a) 1D cut of aniso-TPHAP from 2D GIWAXS and a simulated pattern from the single crystal X-ray analysis. b) The table of peak assignments. The d-spacing of diffractions were calculated with $d = n\lambda/2\sin\theta$. Miller index was placed on the single crystal X-ray analysis: orthorhombic *Pbca*.

a)

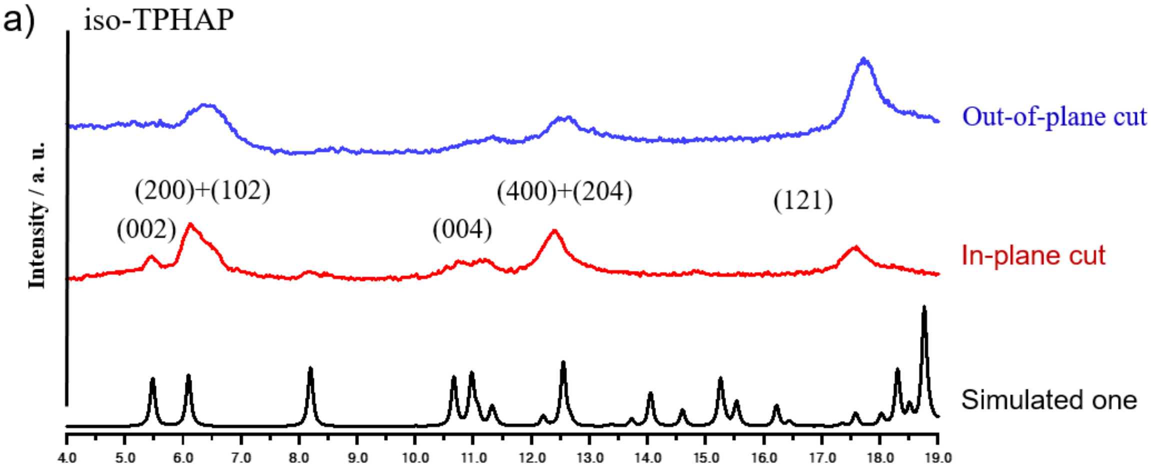

b)

Index	n	2θ (°)	d (Å)
(002)	1	5.46	11.76
(200)+(102)	1	6.11	10.50
(004)	2	11.19	11.48
(400)+(204)	2	12.38	10.38
(121)	1	17.65	3.65

Fig. S15. Assignment of diffractions in GIWAXS of iso-TPHAP. a) 1D cut of iso-TPHAP from 2D GIWAXS and a simulated pattern from the single crystal X-ray analysis. b) The table of peak assignments. The d-spacing of diffractions were calculated with $d = n\lambda/2\sin\theta$. Miller index was placed on the single crystal X-ray analysis: orthorhombic *Pbca*.

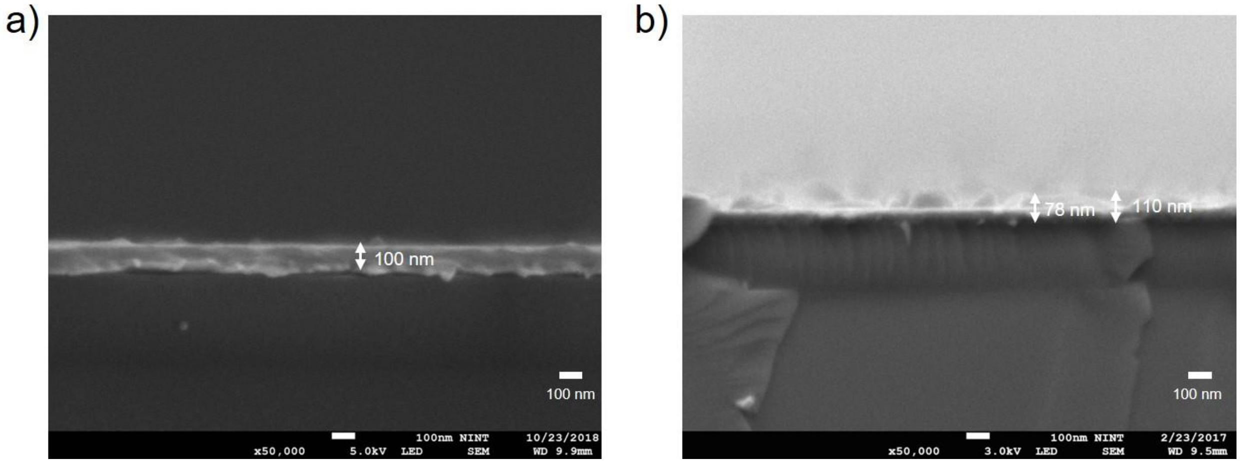

Fig. S16. Cross-sectional FE-SEM images of a) aniso-TPHAP and b) iso-TPHAP.

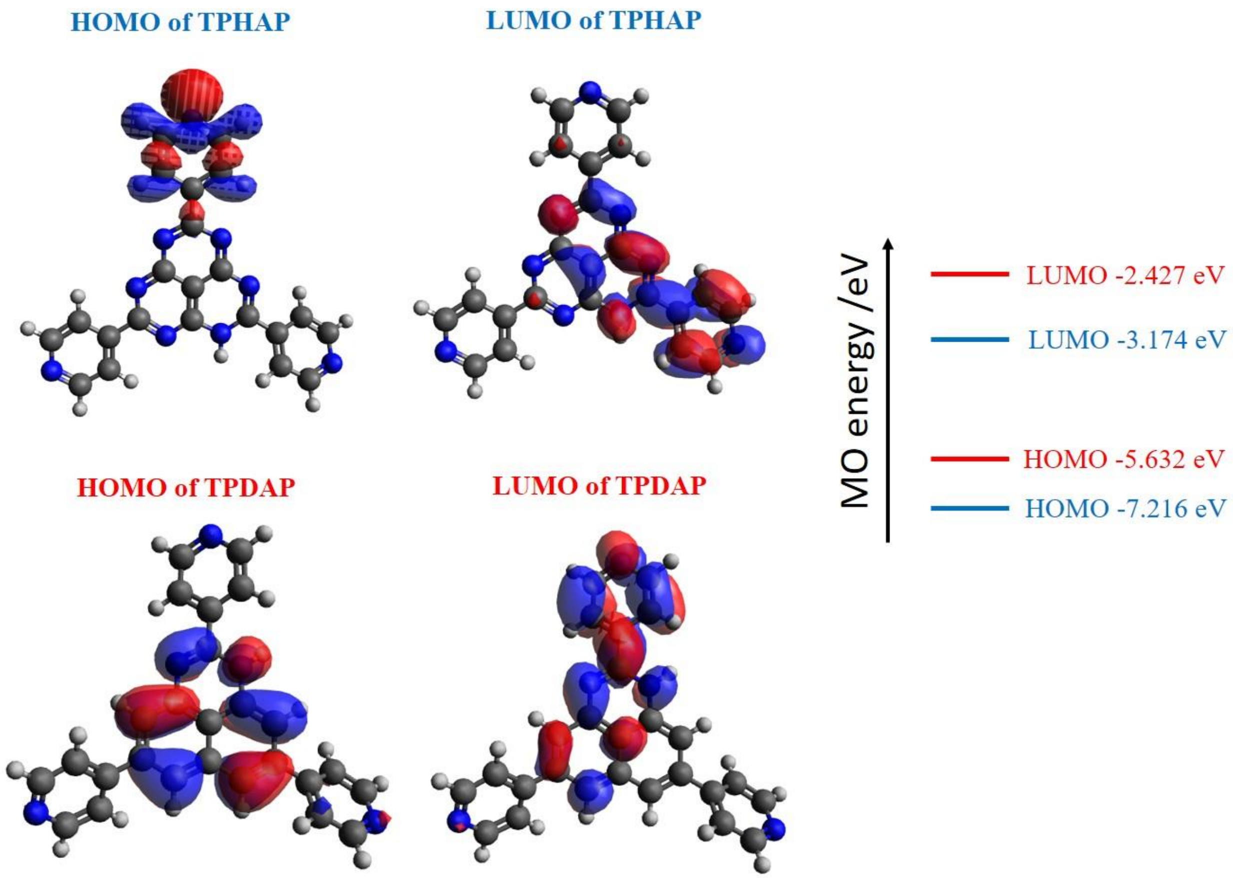

Fig. S17. HOMO-LUMO energy levels of TPDAP and TPHAP. The molecular orbitals of **TPDAP** and **protonated TPHAP** were calculated by the DFT method with the B3LYP/6-31G* level of theory using the Gaussian09 software package.

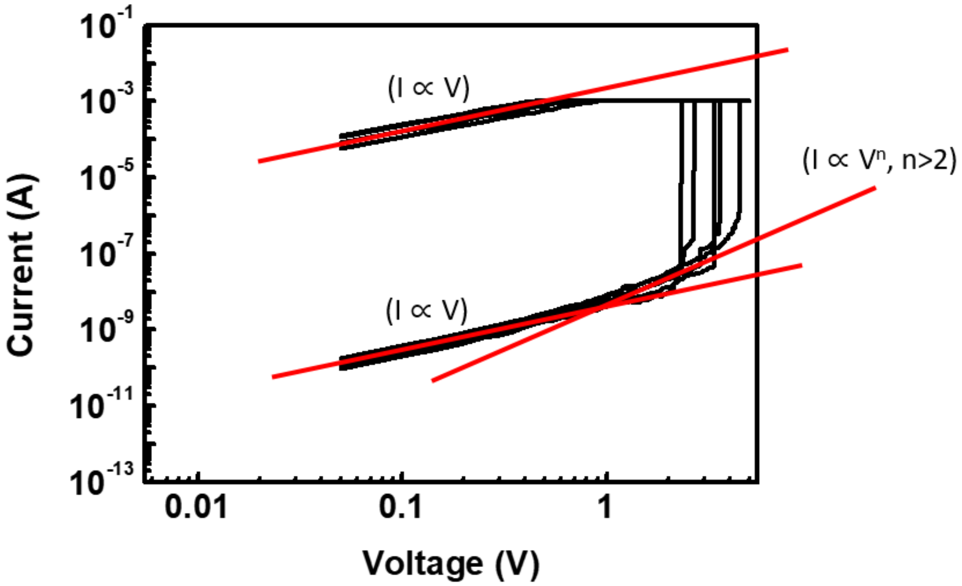

Fig. S18. Double-log plot of I-V characteristic of Au/aniso-TPDAP/Au/SiO_2/Si device. In the low voltage region (<1V), the current is proportional to the applied bias, while a square dependence of current on the applied voltage was observed in the high voltage region (>1V). At the high voltage region, the current was controlled by the traps in the active layer. TPDAP acted as a charge-trapping site. After the trap sites were filled with holes, the Ohmic region appeared as a low resistance state.

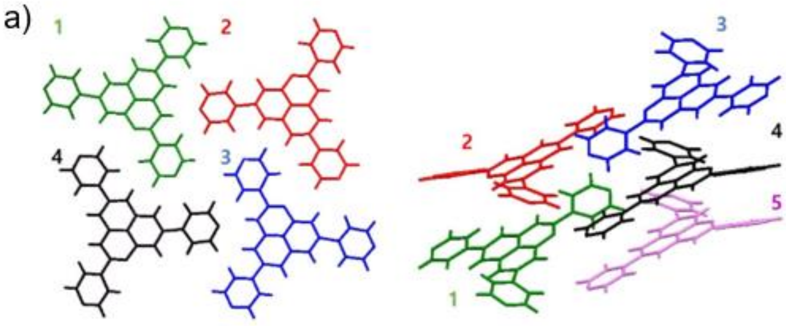

b)

Direction	Transfer integral (eV)
1 -> 2	0.00072
1 -> 3	0.00004
1 -> 4	0.00037
**Mobility (in-plane)**	6.51 x 10^{-6} cm^2/V·s

Direction	Transfer integral (eV)
4 -> 5	0.12098
**Mobility (out-of-plane)**	0.53 cm^2/V·s

Fig. S19. Theoretical calculation for hole mobility in TPDAPs. a) Crystal structure of TPDAP for the calculation of reorganization energy and transfer integrals. The molecular interactions between 1, 2, 3, and 4 are corresponding to the in-plane direction and that between 4 and 5 is corresponding to the out-of-plane direction. b) Calculated hole mobility of in-plane and our-of-plane molecular interaction. The used reorganization energy for mobility calculation was 0.212 eV. The detailed equation for calculation was described in Materials and Methods.

References for ESI

S1 J. Y. Koo, Y. Yakiyama, J. Kim, Y. Morita and M. Kawano, *Chem. Lett.*, 2015, **44**, 1131.

S2 Y. Yakiyama, A. Ueda, Y. Morita and M. Kawano, *Chem. Commun.*, 2012, **48**, 10651.

S3 G. M. Sheldrick, *Acta Cryst.*, 2015, **C71**, 3.
